# Effects of Pyrolysis Temperatures and Modified Methods on Rice Husk-Derived Biochar Characteristics and Heavy Metal Adsorption

**DOI:** 10.3390/molecules30173616

**Published:** 2025-09-04

**Authors:** Zhaoqin Huang, Qin Wang, Yufeng Zhang, Buyun Du, Jun Zhou, Dongliang Ji

**Affiliations:** 1 College of Environmental Ecology, Jiangsu Open University, Nanjing 210017, China; huangzq@jsou.edu.cn (Z.H.); duby@jsou.edu.cn (B.D.); 2 School of Environmental Science and Engineering, Nanjing Tech University, Nanjing 211816, China; wq_917@163.com (Q.W.); zhangyuf99@126.com (Y.Z.); 3 Institute of Soil Science, Chinese Academy of Sciences, Nanjing 210008, China; zhoujun@issas.ac.cn

**Keywords:** biochar, modification, adsorption, Cd (II), Pb (II)

## Abstract

Biochars were prepared from rice husk at different pyrolysis temperatures (300, 400, and 500 °C) and then modified by nitric acid (HNO_3_) and potassium hydroxide (KOH). The chemical and physical properties were characterized, and the adsorption ability of biochars for the removal of Cd (II) and Pb (II) was investigated. The results showed that with increasing pyrolysis temperature, the aromaticity of rice husk biochar increased while its polarity decreased and both specific surface area and total pore volume significantly increased. Both HNO_3_ and KOH modification significantly changed the oxygen-containing functional groups in biochar, especially biochars prepared at lower pyrolysis temperatures. HNO_3_ modification introduced nitro and carboxyl groups on the surface of HNO_3_-BC300, increasing the ether bond functional groups, while KOH modification increased the content of hydroxyl groups on KOH-BC300 and reduced the ether bond groups. At the same time, the modification of rice husk-derived biochar greatly enhanced the ability to absorb Cd (II) and Pb (II) from aqueous solution. Notably, KOH-BC300 exhibited the highest adsorption capacities, reaching 72.14 mg·g^−1^ for Cd (II) and 170.84 mg·g^−1^ for Pb (II). These results demonstrate that KOH modification was more effective than HNO_3_ modification at enhancing the adsorption of Cd (II) and Pb (II) onto rice husk-derived biochar. In addition, the specific surface area and total pore volume of biochar increased significantly after HNO_3_ and KOH modification. It was concluded that biochar’s adsorption performance might be greatly improved by increasing its oxygen-containing functional groups and specific surface area, but the effect of oxygen-containing functional groups was greater than that of specific surface area. Thus, KOH-modified biochar (KOH-BC300) can be used as an effective sorbent for heavy metal removal from wastewater.

## 1. Introduction

Heavy metals (HMs) have been released into soil and water environments due to industrial effluent discharge, smelting and mining activities, agricultural runoff, urban and domestic waste, atmospheric deposition, and geological weathering. This has led to HM pollution all over the world. HMs are of substantial concern owing to their non-biodegradable and bio-accumulative toxicity in living organisms [1,2]. As typical heavy metal pollutants, cadmium (Cd) and lead (Pb), which were commonly used in printing, electroplating, and textile manufacturing, are ubiquitous in wastewater and considered to be toxic to plants, animals, and humans. Thus, there is a critical need for efficient and economically viable methods to decrease heavy metal concentrations or restrict their presence and mobility in wastewater.

HMs can be removed by adsorption, chemical precipitation, ion exchange, and membrane filtration from wastewater. Among these technologies, adsorption has been considered to be the most practical technology for HM removal in wastewater in recent years due to its ease of operation, eco-friendliness, high efficiency, and low cost [3,4,5,6]. Biochar, the carbonaceous material produced from various organic feedstocks under thermal pyrolysis with limited oxygen, has received increasing research attention because of its unique features such as rich carbon content, large specific surface area, high cation exchange capacity, and stable structure in many environmental areas, and is being investigated for HM removal in wastewater [7,8,9]. The adsorption capacity of biochar depends on its physiochemical properties, while physiochemical properties vary with the pyrolysis conditions and feedstocks. The choice of a suitable pyrolysis variant and biochar modification method is vital to improve the adsorption capacity of biochar [10,11,12]. Chemical modification is the most widely used method, which can modify biochar by acid, alkali, metal salt or oxidizing agents, carbonaceous materials, and so on [13]. Acid modification can eliminate impurities like metals and introduce acidic functional groups on biochar’s surface. Additionally, it can alter the surface area, with the impact varying depending on acid type, concentration, feedstock, and production conditions. Alkaline modification, primarily employing potassium hydroxide and sodium hydroxide as activating agents, aims to enhance biochar’s specific surface area and introduce oxygen-containing functional groups. Jin et al. found that potassium hydroxide modification increased the surface area of pyrolysis-derived municipal solid waste biochar, thereby improving As(V) adsorption [14]. However, Sun et al. demonstrated that potassium hydroxide modification decreased the surface area of wheat straw-derived biochar produced via hydrothermal carbonization [15]. These results showed that the effect of alkaline modification on biochar’s surface area also depended on the types of feedstock and preparation methods.

Many kinds of biomass, including agricultural waste, forestry waste, and manure, could be used for biochar preparation [16,17,18]. Rice husk is a plentiful resource in Asian countries, but its use is now extremely limited [19]. It is a kind of low-moisture-content material that can be pyrolyzed directly, and its high carbon content helps in converting it into energy-rich biochar after thermochemical treatment [20]. Moreover, the use of rice husk waste to produce biochar for wastewater treatment will remarkably reduce the total expenses associated with wastewater and solid waste treatment [17]. However, only a few studies have examined modified rice husk biochars and their environmental applications.

In this study, rice husk biochars were pyrolyzed at 300 °C, 400 °C, and 500 °C and then modified by nitric acid (HNO_3_) and potassium hydroxide (KOH). Adsorption kinetics and adsorption thermodynamics were used to evaluate the adsorption capacity. This study mainly investigated the influence of modification conditions and pyrolysis temperature on the characteristics of rice husk-derived biochar, and correlated it with its adsorption performance for HMs to explore its adsorption mechanism. The specific objectives are as follows: (1) prepare and characterize unmodified and modified rice husk biochars; (2) compare the sorption capacities of these biochars to remove Cd and Pb from aqueous solution and investigate their sorption mechanism further; (3) select the optimal biochar properties and modification methods for removing heavy metals from wastewater more efficiently.

## 2. Results and Discussion

### 2.1. Thermogravimetry

**Figure 1:**
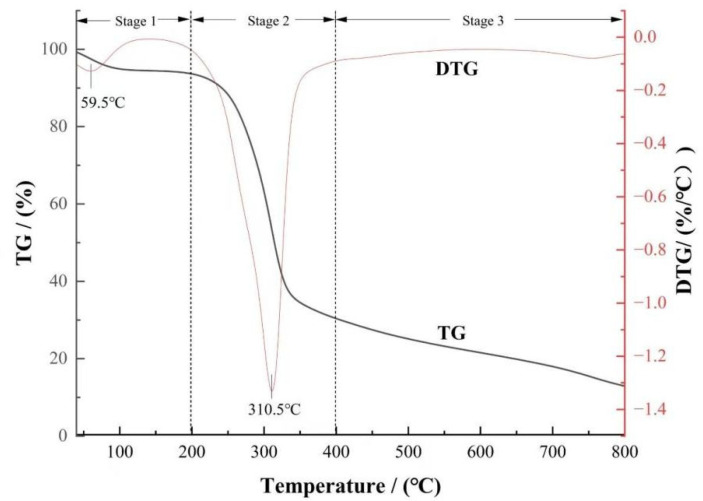
TG and DTG curves of rice husk biomass.

The thermogravimetric curve (TG curve) and its derivative curve (DTG curve) of rice husk biomass during pyrolysis at a heating rate of 20 °C·min^−1^ are shown in Figure 1. The total mass loss of rice husk at the final pyrolysis temperature was 70.77 %, which was mainly attributed to its higher volatile content and lower ash content. The pyrolysis process of rice husk can be divided into three stages: the initial weight loss peak in the first stage was the removal of water from the rice husk (<10%), the second stage was the removal of the main volatiles, and the third stage was the continuous slight decomposition of macromolecular substances. These three stages are the main stages in pyrolysis.

Pyrolysis kinetic analysis showed that the main weight loss stage (200~400 °C) contributed 56.86% of the mass loss, accounting for 80.35% of the total weight loss [21]. This process was mainly due to hemicellulose depolymerization (220~315 °C), cellulose degradation (315~400 °C), and partial lignin cracking. Among them, hemicellulose was preferentially decomposed at lower temperatures due to its amorphous structure and multi-branched chain characteristics; the linear polymer characteristics of cellulose endow it with high thermal stability. Due to its complex aromatic ring structure, the decomposition temperature of lignin shows a wide distribution (200~800 °C) [22,23]. In the secondary stage (>400 °C), the weight loss only accounted for 13.91%, which mainly involved the aromatic ring recombination of residual lignin and the graphitization process of the carbon matrix. The slow decomposition kinetics were regulated by the steric hindrance effect of high-molecular-weight polymers [24].

It is worth noting that the temperature range of 300~500 °C included the pyrolysis phase transition process of the main components of rice husk biomass: hemicellulose was completely decomposed at 300 °C, and cellulose decomposition entered the end stage (400 °C termination), while lignin still maintained continuous depolymerization in this interval. This thermodynamic property showed that the preparation of biochar in this temperature zone can not only ensure the full carbonization of raw materials (volatile removal rate > 80%) but also avoid the collapse of pore structures caused by excessive temperature, which provided a scientific basis for regulating the balance between the surface functional groups and pore structure of rice husk biochar.

### 2.2. Elemental Analysis

**Figure 2:**
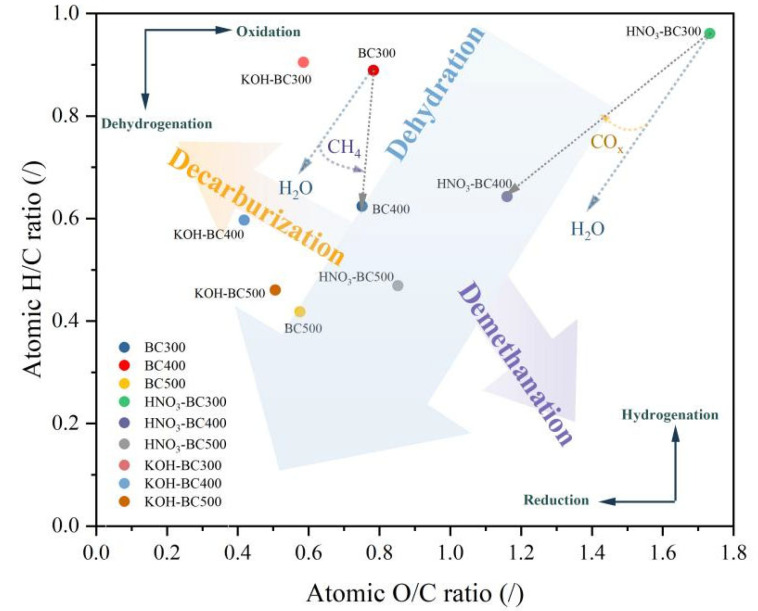
Van Krevelen diagram of atomic H/C and atomic O/C ratios for pristine and modified biochar.

Pyrolysis temperature and modification methods considerably influenced the elemental composition of biochar (Table 1). As the pyrolysis temperature increased from 300 to 500 °C, the C content of rice husk-derived biochar increased from 46.96% to 55.05%, while the H and O content decreased from 3.50% to 1.93% and 48.99% to 42.23%, respectively. This indicated that carbonization, dehydrogenation, and deoxidation reactions occurred with the increase in pyrolysis temperature. The molar ratios of H/C and O/C were utilized to evaluate the polarity of the biochar and the degree of carbonization. When the pyrolysis temperature rose from 300 to 500 °C, the molar ratios of H/C and O/C decreased from 0.89 to 0.42 and 0.36 to 0.09, respectively, indicating that high temperature promoted the aromatization reaction of the rice husk biochar, increased the aromaticity, and decreased polarity. Based on previous research, the removal of oxygen in the form of CO_2_ would result in the molecular formula in the van Krevelen diagram approaching H/C = 1. Conversely, the composition would alter to approach H/C = 0 if oxygen were removed in the form of water [25]. The Van Krevelen diagram (Figure 2) shows that the H/C decline conformed to the dominant path of the dehydration reaction (H_2_O removal makes H/C approach 0) with the pyrolysis temperature increase.

Different modification methods variably affected the elemental contents. HNO_3_ modification notably increased the N and O contents by 3.13~4.70 and 1.74~3.16 times, respectively, compared to unmodified biochar prepared under identical pyrolysis temperature. This could be due to the HNO_3_ oxidation of biochar resulting in the introduction of functional groups containing oxygen, such as nitro groups and carboxyl on the surface [26], thus enhancing the hydrophilicity of biochar [19]. The KOH treatment of rice husk-derived biochar enhanced the contents of C, O, and H. This finding indicated that KOH modification could also increase the oxygen-containing functional groups and polarity. These changes in rice husk biochar are different from those in biochar prepared from bamboo and sludge [14,27]. KOH treatment produced a lower ratio of O/C compared to HNO_3_-modified biochars, which indicated the greater hydrophobicity and aromaticity of KOH-modified rice husk-derived biochar. Furthermore, pristine biochar’s ash content exhibited an increase with elevated pyrolysis temperatures, suggesting that mineral elements were formed and accumulated within the rice husk biochar during pyrolysis [28]. After modification, the ash content was decreased. The KOH modification reduced ash content by 30.66–58.65%, which was attributed to the reaction of KOH with SiO_2_ to form soluble K_2_SiO_3_ [29]. However, the decrease in HNO_3_-modified ash was smaller, which may be related to its selective dissolution of metal oxides.

### 2.3. BET and Pore Analysis

**Figure 3:**
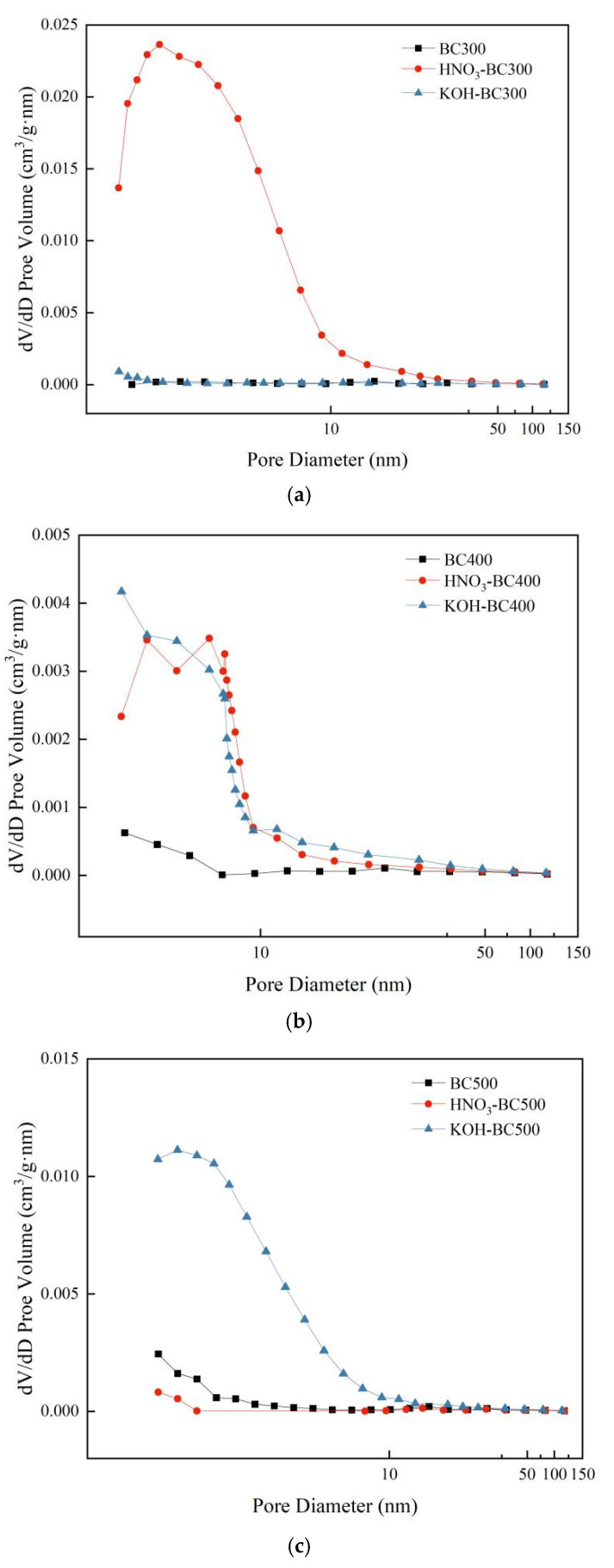
Pore size distribution of pristine and modified biochar. (**a**–**c**) show the pore size distribution of biochar prepared at 300 °C, 400 °C, and 500 °C, respectively, both before and after modification.

Pyrolysis temperature and modification methods significantly influenced the specific surface area, pore volume, pore size, and pore size distribution of biochar, as shown in Table 2 and Figure 3. The specific surface area and total pore volume of pristine biochar increased significantly with the increase in pyrolysis temperature, while the average pore size decreased significantly. Specifically, with the increase in pyrolysis temperature from 300 °C to 500 °C, the specific surface area of unmodified biochar increased from 0.6 m^2^·g^−1^ to 23.0 m^2^·g^−1^, the total pore volume increased from 0.0089 cm^3^·g^−1^ to 0.021 cm^3^·g^−1^, and the average pore size decreased from 61.50 nm to 3.58 nm. This shows that high-temperature (≥400 °C) pyrolysis can significantly promote the development of micropores and optimize the pore distribution [30].

After modification, different modification reagents showed significant differences in the regulation of biochar pore structure. HNO_3_ modification increased the specific surface area of biochar (119.1 m^2^·g^−1^) at 300 °C, which was 199 times higher than that of unmodified samples prepared at the same temperature, but the modification effect decreased sharply with the increase in temperature, revealing the specific activation of acid etching on the amorphous structure of low-temperature biochar [31]. It is worth noting that the specific surface area and total pore volume of HNO_3_-BC500 were smaller than those of BC500, while the average pore size was larger. This is because the average pore size of BC500 is small, and the HNO_3_ modification on this basis will cause some smaller and loose pores to collapse [32,33], thus merging to form a large pore structure. KOH modification showed typical high-temperature activation characteristics. With the increase in preparation temperature from 300 °C to 500 °C, the specific surface area of KOH-modified biochar increased from 3.0 m^2^·g^−1^ to 74.9 m^2^·g^−1^, the total pore volume increased from 0.0091 cm^3^·g^−1^ to 0.065 cm^3^·g^−1^, and the average pore size decreased from 11.48 nm to 3.45 nm. This indicated that KOH modification could etch and remove the disordered and amorphous carbon structure in biochar at high temperatures so as to optimize the pore structure and retain the carbon skeleton with a high graphitization degree. Thus, the pore structure of biochar was significantly improved [30]. The pore size distribution of biochar prepared at different temperatures before and after modification was also consistent with these findings (Figure 3). HNO_3_ modification significantly changed the pore structure of rice husk-derived biochar prepared at 300 °C and promoted the formation of a large number of small-sized pores, making small-sized pores the main pore structure contributor. Both KOH and HNO_3_ modification can make biochar prepared at 400 °C form a small-sized pore structure. In contrast, KOH modification significantly increased the small pore volume of biochar prepared at 500 °C.

### 2.4. SEM Analysis

**Figure 4:**
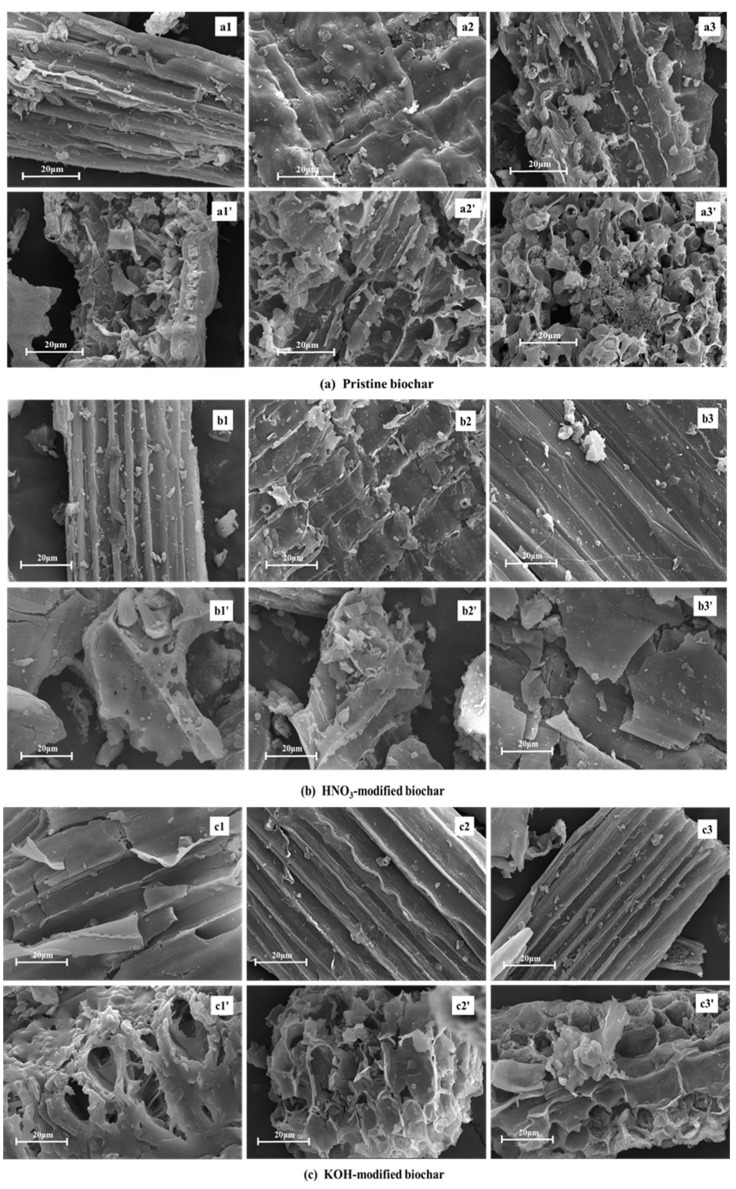
SEM analysis of pristine and modified biochar. (**a**) Pristine biochar: (**a1**,**a1′**), (**a2**,**a2′**) and (**a3**,**a3′**) show the SEM images of the inner (outer) BC300, BC400, and BC500, respectively; (**b**) HNO_3_-modified biochar: (**b1**,**b1′**), (**b2**,**b2′**) and (**b3**,**b3′**) show the SEM images of the inner (outer) HNO_3_-BC300, HNO_3_-BC400, and HNO_3_-BC500, respectively; (**c**) KOH-modified biochar: (**c1**,**c1**′), (**c2**,**c2′**) and (**c3**,**c3′**) show the SEM images of the inner (outer) KOH-BC300, KOH-BC400, and KOH-BC500, respectively.

Pyrolysis temperature and modification methods also influence the surface morphology of biochar. SEM images (Figure 4) visually demonstrated these effects, further verifying the above results. Since the raw rice husk has two surfaces (inner and outer), the rice husk-derived biochar has a smooth interior concave surface and a rough exterior convex surface [34]. The inner and outer surface morphology of unmodified biochar was rough, and the pore distribution was irregular (Figure 4a). However, the inner surface morphology of biochar modified by HNO_3_ became relatively smooth, and the vertical structure became clearly visible (Figure 4b). This HNO_3_ modification decomposed part of the carbon structure through acidic erosion, which significantly promoted the formation of micropores and mesopores, thereby optimizing the specific surface area and pore structure. Furthermore, the KOH-modified biochar showed the smoothest inner surface and the clearest hierarchical vertical structure among the three modified samples (Figure 4c), which proved that KOH modification was more inclined to corrode the carbon fragments, so as to open the closed pores.

### 2.5. FTIR Analysis

**Figure 5:**
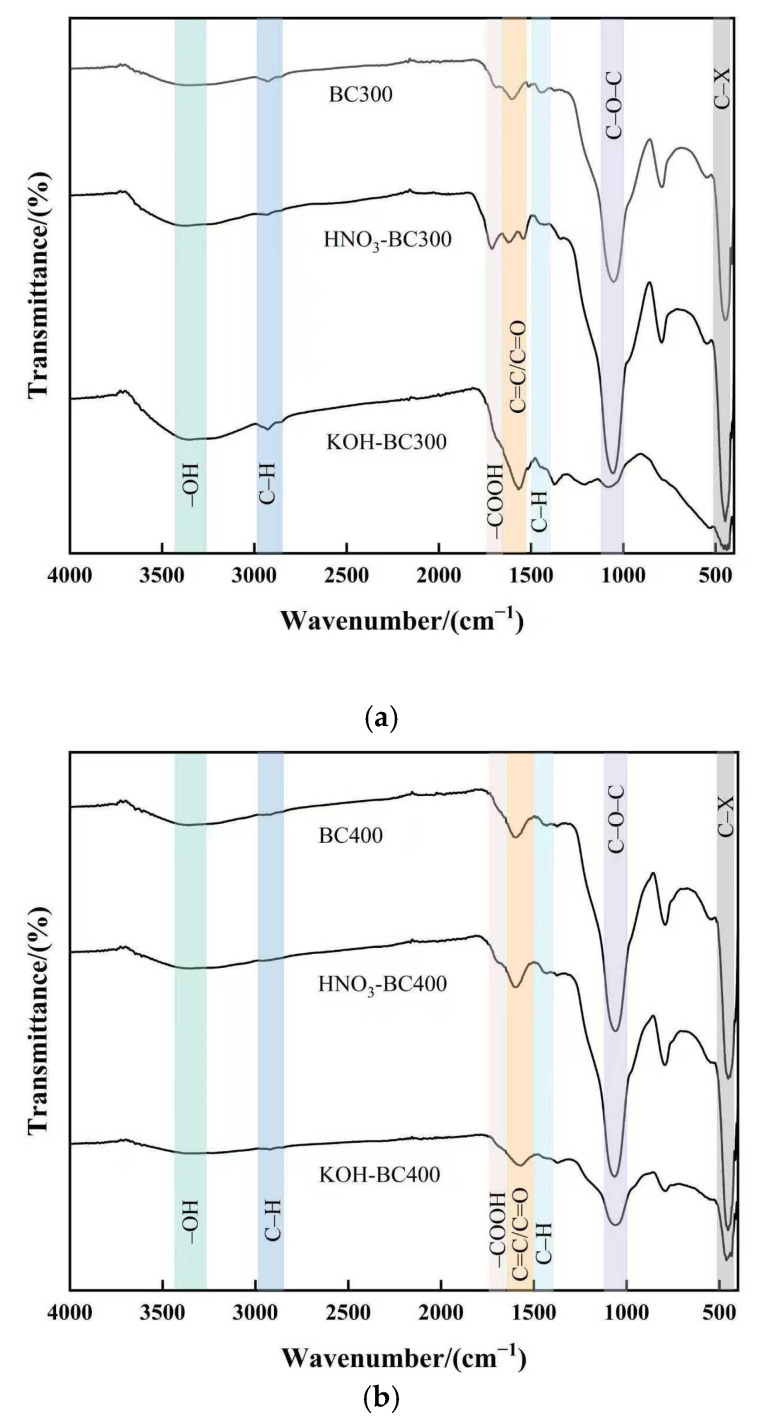

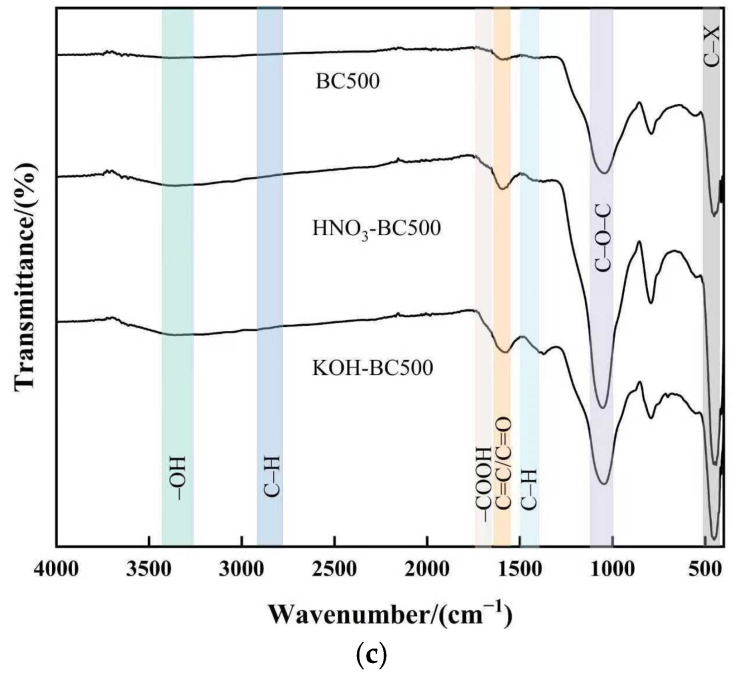
FTIR spectra of pristine and modified biochar at different pyrolysis temperatures. (**a**–**c**) show the spectra of biochar prepared at 300 °C, 400 °C, and 500 °C, respectively, both before and after modification.

The FTIR spectra of all pristine and modified biochars are presented in Figure 5. As can be seen in Figure 5, the FTIR spectra of biochar had characteristic peaks at 3290 cm^−1^, 2920 cm^−1^, 1490 cm^−1^, 1712 cm^−1^, 1606 cm^−1^ and 1058 cm^−1^, which were attributed to the –OH stretching of hydroxyl groups, antisymmetric –CH_2_ stretching, C=O stretching of carboxyl and ketones, C=C stretching of aromatic components, a small amount of C=O stretching in quinines and ketonic acids, and the symmetric stretching vibration of C–O–C or C–O, respectively [35]. The pyrolysis temperature barely affected the type of functional groups. However, it did influence the strength of the characteristic peak to some extent. The –OH stretching vibration which represented the presence of cellulose decreased with increasing pyrolysis temperature and nearly vanished at 500 °C, indicating that the cellulose was almost completely degraded at this temperature. The stretching vibration of –CH_2_ was more obvious at the pyrolysis temperature of 300 °C. This showed that aliphatic groups disappeared from biochar at higher pyrolysis temperatures. Organic aliphatic hydrocarbons degraded into methane, carbon dioxide, and other gases, while aromatic structures formed [36]. The C=C stretching vibration is typically found between 1700 and 1610 cm^−1^. However, the peaks shifted to a lower frequency of 1606 cm^−1^, as the aromatic ring was connected with a C=C double bond due to the conjugate effect. These findings suggested that with the pyrolysis temperature increasing, the oxygen-containing functional groups of biochar decreased and the aromaticity increased, which was consistent with the decrease in H/C and O/C ratios in element analysis.

Modification influences the type and number of functional groups [37]. After HNO_3_ modification, the peak intensity at 1606 cm^−1^ and 1050 cm^−1^ of biochar prepared at different temperatures increased, indicating that HNO_3_ modification increased the number of oxygen-containing groups such as C=C/C=O and C–O–C/C–O in biochar. In addition, for HNO_3_-BC300, the peak intensity at 1712 cm^−1^ increased, which meant that the carboxyl groups increased in HNO_3_-BC300. It was worth noting that HNO_3_-BC300 produced new characteristic peaks at 1560 cm^−1^ and 1330 cm^−1^, representing the asymmetric and symmetric vibrations of –NO_2_, respectively. The emergence of new peaks was mainly due to the incomplete carbonization of biochar prepared at this temperature, which formed surface groups containing nitro and nitrate under the oxidation of HNO_3_. After KOH modification, the C–O–C or C–O symmetric stretching vibration peaks of KOH-BC300 and KOH-BC400 at 1050 cm^−1^ and the C=O stretching vibration peaks of carboxyl and ketone groups at 1712 cm^−1^ were weakened. This may be due to the decarboxylation reaction caused by KOH modification, which weakens the stretching vibration band of C–O–C groups. For biochar prepared at a lower temperature (BC300), the intensity and breadth of the peak produced by the –OH bond stretching vibration at 3290 cm^−1^ were enhanced due to the introduction of additional hydroxyl groups after KOH modification. At the same time, the stretching vibration of C=C on the aromatic ring and C=O stretching in quinines and ketonic acids of KOH-BC300 and KOH-BC500 increased, indicating that the aromaticity of these biochars modified by KOH increased, while the KOH-BC400 showed the opposite results. In order to study the changes and fate of C, O, and N atoms, we used XPS to analyze the functional groups of biochar further.

### 2.6. XPS Analysis

**Figure 6:**
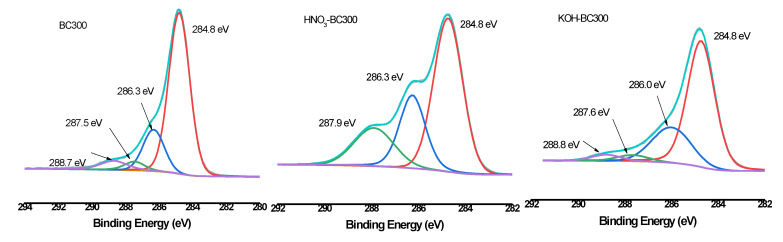

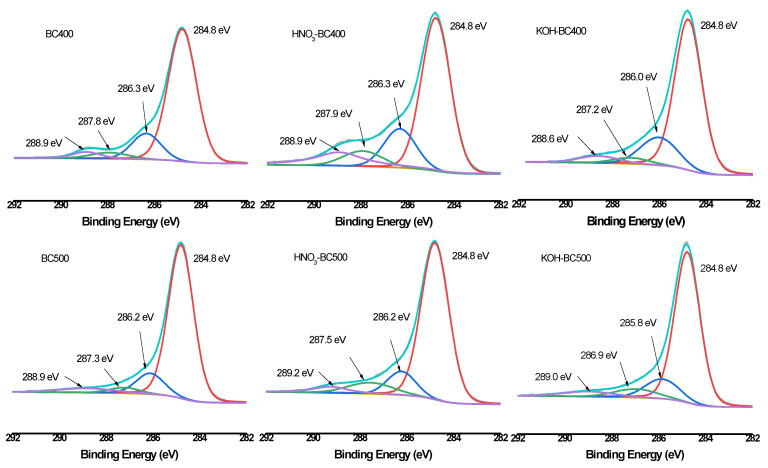
XPS C1s scan and peak fitting for pristine and modified biochar at different pyrolysis temperatures.

**Figure 7:**
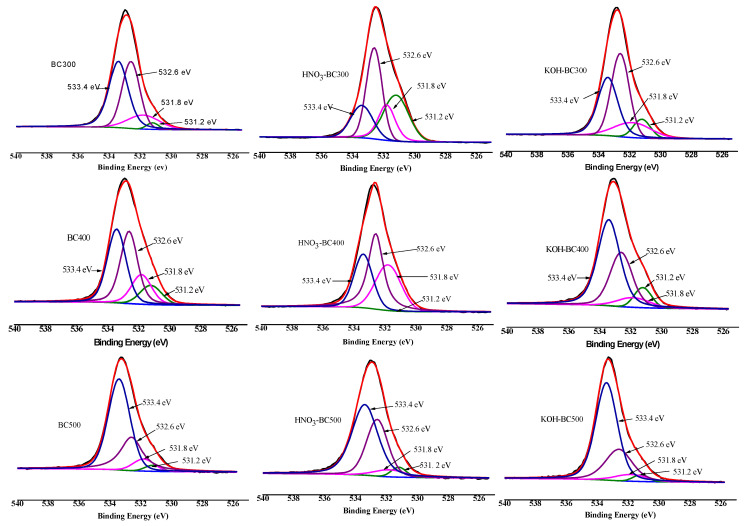
XPS O1s scan and peak fitting for pristine and modified biochar at different pyrolysis temperatures.

**Figure 8:**
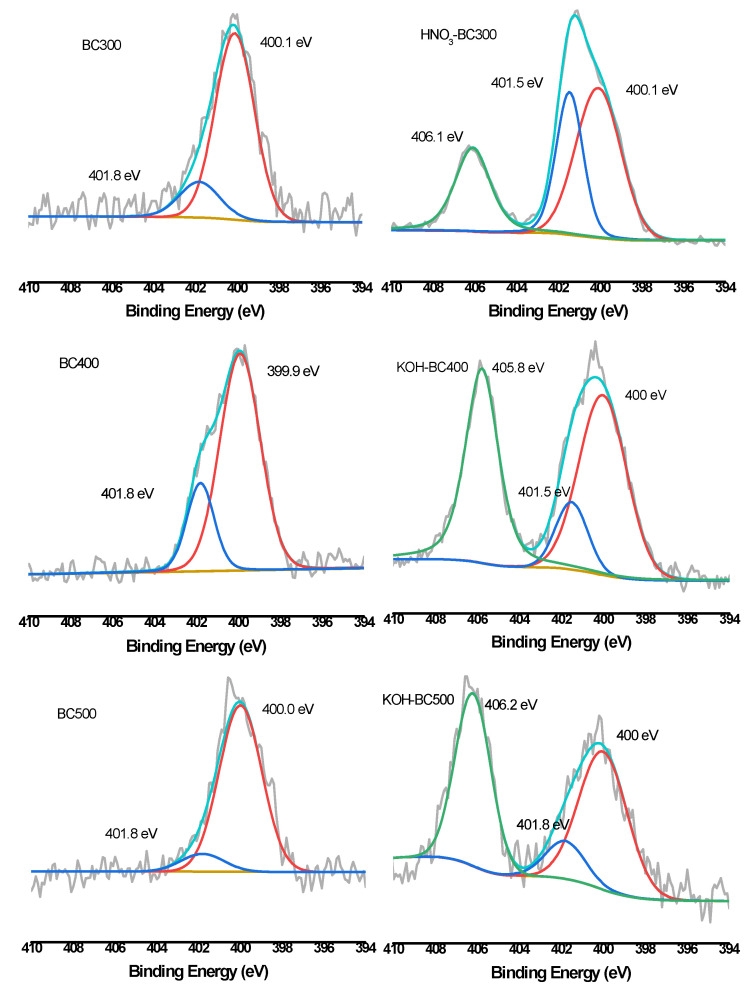
XPS N1s scan and peak fitting for pristine and HNO_3_-modified biochar.

XPS was then employed to quantify the different C, O, and N forms present on the surface of biochar. As shown in Figure 6, Figure 7 and Figure 8 and Table 3, the relative atomic percentages of C, O, and N and their chemical bonding states were revealed for pristine and modified biochar at different pyrolysis temperatures. According to published articles, the C1s, O1s, and N1s spectra were deconvoluted as follows [38,39,40]. The typical C1s spectra of biochar were split into four peaks. As indicated, carbon was bound to carbon and hydrogen (C–C(H), C=C, 284.8 eV), carbon was bound to oxygen or nitrogen (i.e., alcohols, amines, or amides, C–(O,N), 285.8–286.3 eV), carbon formed two single bonds or one double bond with oxygen (i.e., hemiacetals, acetals, amides, and carboxylates, C=O, O–C–O, 286.9–287.9 eV), and carbon formed one double bond and one single bond with oxygen (i.e., carboxyl or ester functionalities, O–C=O, 288.6–289.2 eV). Similarly, the typical O1s spectra of biochar were deconvoluted into four peaks, as shown in Figure 7. The main O-containing species in the biochar product were carbonyl oxygen of quinines (C=O, 531.2 eV), and oxygen formed single bonds with hydrogen or carbon (i.e., alcohols and ethers, C–O–C, C–OH, 532.6 eV), as well as oxygen atoms in ester or carboxyl groups (O=C–O–(C,H), 531.8, 533.4 eV). The N1s binding energies 399.9–400.1 eV, 401.5–401.8 eV, and 405.8–406.2 eV were assigned as N–C, N–C–COOH, NH_4_^+^ (ammonium-N), and nitrate N. The binding energies and their atomic percentages are listed in Table 3.

The O=C–O–(C,H) groups were the dominant O-containing species in the biochar product with several C–O–C, C–OH, and C=O groups. After the introduction of HNO_3_, the O-containing species of the biochar were characterized by a notable change. The C–O–C, and C–OH groups replaced the O=C–O–(C,H) groups to become the major O-containing species of HNO_3_-BC300 and HNO_3_-BC400. The proportion of O atoms on the surface of HNO_3_-BC400 and HNO_3_-BC500 in the form of C–O–C increased, indicating that more ether oxygen-containing groups were produced after modification, which was consistent with the increase in the relative strength of C–O–C groups in the infrared spectrum. In addition, the percentage of functional groups in the form of O=C–O–(C,H) at 533.4 eV in HNO_3_-BC400 and HNO_3_-BC500 decreased, which meant that the carboxyl or ester oxygen-containing functional groups on the surface of biochar decreased after modification, which was also consistent with the results of infrared spectrum analysis. The increase in oxygen-containing functional groups such as C=O at 531.2 eV and O=C–O–(C,H) at 532.8 eV in HNO_3_-BC300 will play an important role in the adsorption and removal of heavy metals. The N atoms in rice husk-derived biochar mainly exist in the form of N–C and N–C–COOH. The appearance of nitrate groups at 406 eV indicated the successful introduction of nitro groups after HNO_3_ modification. By contrast, the variation in biochar prepared at different temperatures after KOH modification did not show the same. The O1s bonding state and relative atomic percentage of biochar surface (Table 3) showed that the proportion of O atoms on the surface of KOH-BC400 and KOH-BC500 in the form of O=C–O–(C,H) at 533.4 eV increased, while that on the surface of KOH-BC300 decreased, but the C=O and C–OH groups increased, which resulted in an overall increase in oxygen-containing functional groups. Chen et al. found that KOH would react with oxygen-containing groups such as –COOH to generate H_2_, CO, CO_2_, CH_4_ and K_2_CO_3_, particularly at lower temperatures [41]. It was speculated that alkali modification would likely induce a decarboxylation reaction on rice husk-derived biochar prepared at a lower temperature (BC300).

### 2.7. Adsorption Kinetics

**Figure 9:**
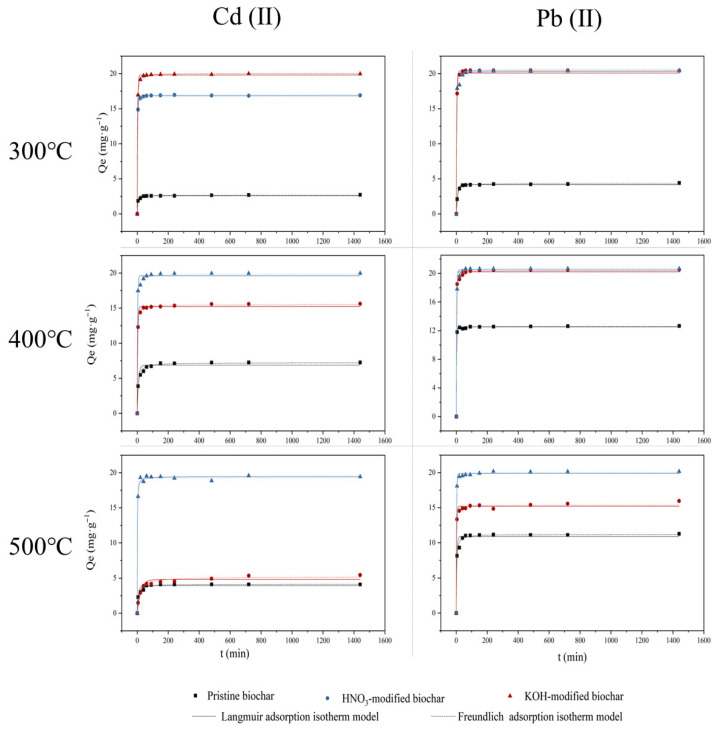
Adsorption kinetics of Cd(II) and Pb(II) by pristine biochar (◼), HNO_3_-modified biochar (●), and KOH-modified biochar (▲).

Figure 9 shows the adsorption kinetics of Cd(II) and Pb(II) (50 mg/L) by pristine biochar and modified biochar at pH = 5.0 ± 0.2 and T = 298 K. Table 4 and Table 5 present the kinetic parameters for the adsorption of Cd(II) and Pb(II), respectively. The k^2^ value indicates the time required for the biochar to achieve equilibrium during the adsorption process. The results demonstrated that either HNO_3_ or KOH modification slightly prolonged the equilibrium time for biochar adsorption but notably enhanced its adsorption capacity. In comparison to the pseudo-first-order kinetic model (R^2^ > 0.9250), the adsorption capacity of each biochar aligned more consistently with the pseudo-second-order kinetic model (R^2^ > 0.9750). This suggested that both Cd (II) and Pb (II) adsorption on different biochars might be mainly chemical adsorption. It may involve chemisorption by sharing forces related to valence or electron exchange [42].

### 2.8. Adsorption Isotherm

**Figure 10:**
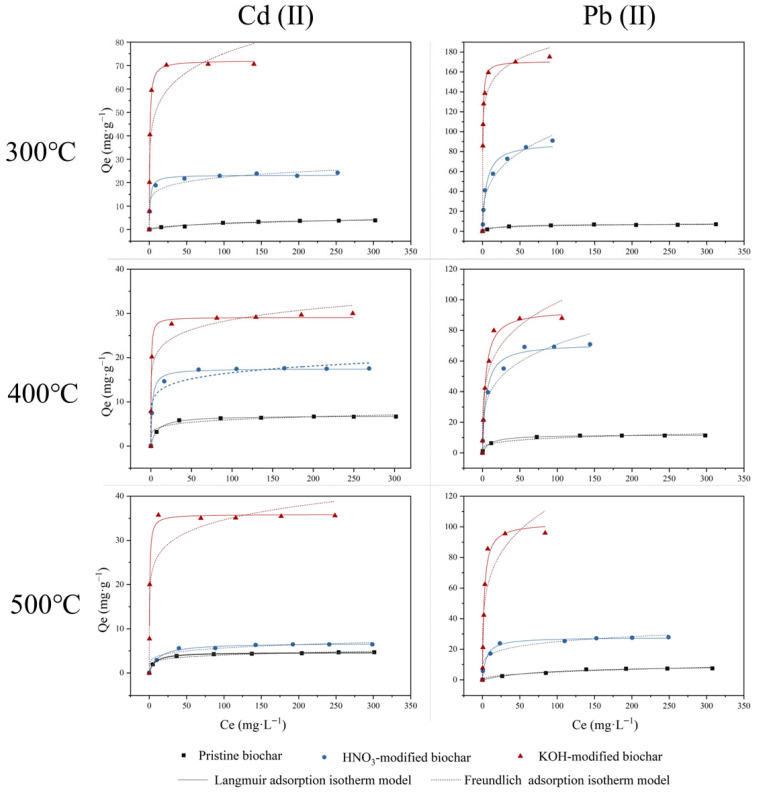
Adsorption isotherm for adsorption of Cd(II) and Pb(II) by pristine biochar (◼), HNO_3_-modified biochar (●) and KOH-modified biochar (▲).

The Langmuir model describes adsorption on homogeneous surfaces, whereas the Freundlich model is better for heterogeneous surfaces [43]. Figure 10 shows the adsorption isotherms of Cd(II) and Pb(II) by unmodified and modified biochar at pH 5.0 ± 0.2 and 298 K. The fitting parameters for Cd(II) and Pb(II) adsorption are summarized in Table 6 and Table 7. Compared to Cd(II), a higher affinity for Pb(II) appeared in all pristine and modified biochars, which could be explained by the contrasting chemical properties of the two metals. Pb(II) had a greater atomic weight, ionic radius, and electronegativity than Cd(II). These differences indicated that Pb(II) was more likely to be adsorbed through inner sphere surface complexation or sorption reactions, making it more favorable for biochar adsorption [44]. The results (Table 6 and Table 7) showed that both the Langmuir model and Freundlich model fitted the adsorption isotherm well with R^2^ values higher than 0.8141, with the Langmuir model (R^2^ ≥ 0.9230) fitting better than the Freundlich model (R^2^ ≥ 0.8141). Thus, the modeling results confirmed that the surfaces of rice husk-derived biochars were more homogeneous and the adsorption on the biochar was not a strict monolayer physical adsorption process, but might involve some chemical interactions. The adsorption efficacy of biochar after modification was significantly improved. The results of the equation-fitting analysis demonstrated that the maximum adsorption capacity for Cd(II) and Pb(II) of HNO_3_-modified biochar was enhanced relative to that of pristine biochar, with an increase ranging from 1.45 to 3.87 and 2.78 to 12.52 times, respectively. The adsorption effectiveness of HNO_3_-BC300 for Cd(II) and Pb(II) was found to be effective at 23.12 mg·g^−1^ (R^2^ = 0.9264) and 89.46 mg·g^−1^ (R^2^ = 0.9653). Biochar modified by KOH exhibited superior adsorption efficacy for Cd(II) and Pb(II), which increased by approximately 4.24~12.10 times and 7.89~23.92 times compared to unmodified biochar, respectively. Furthermore, KOH-modified biochar in this study exhibited exceptional adsorption effectiveness for Cd(II) and Pb(II), surpassing that of adsorbents documented in some other studies (Table 8), with 72.14 mg·g^−1^ for Cd(II) and 170.84 mg·g^−1^ for Pb(II).

### 2.9. Adsorption Mechanisms

The Q_m_ values of heavy metals on modified biochars were higher than unmodified ones, which may be due to the increasing specific surface area and binding sites associated with oxygen-containing functional groups, such as hydroxyl, carboxyl, carbonyl, ether bond groups, and so on. In addition, the adsorption capacity of KOH-modified biochar increased more than HNO_3_-modified biochar, indicating that KOH was more suitable for the modification of rice husk biochar. Biochar can adsorb heavy metals through surface complexation, electrostatic adsorption, cation-Π interaction, ion exchange, and adsorption co-precipitation [44,50,51]. Generally, the specific surface area was proportional to the adsorption performance of biochar, which provided the adsorption sites and pollutant diffusion channels [52]. In this work, HNO_3_-BC300 had the largest specific surface area, which was 206 and 41 times larger than that of BC300 and KOH-BC300, respectively. The maximum adsorption capacities for Cd (II) and Pb (II) of HNO3-BC300 were 23.12 mg·g^−1^ (R^2^ = 0.9264) and 89.46 mg·g^−1^ (R^2^ = 0.9653), respectively. However, the KOH-BC300 exhibited the highest adsorption capacity, with values of 72.14 mg·g^−1^ (R^2^ = 0.9791) for Cd(II) and 170.84 mg·g^−1^ (R^2^ = 0.9788) for Pb(II). Although HNO_3_-BC300 had the largest specific surface area, the maximum adsorption capacity of Cd(II) and Pb(II) was lower than that of KOH-BC300, because according to the FTIR and XPS spectra, the –OH functional group was introduced into KOH-BC300, the amount of C=O functional groups increased, and the C–O–C functional groups decreased. Generally, the ether bond C–O–C has weak interactions with metal ions due to the low electron cloud density; thus, the reduction of C–O–C groups on the surface of biochar helps expose more micropores and edge active sites, enhancing the contact efficiency of heavy metal ions with adsorption sites. As the key oxygen-containing functional groups in biochar, C=O and –OH groups can form stable surface complexes with heavy metal ions through coordination and can also adsorb heavy metals through ion exchange and electrostatic attraction. Therefore, with an increase in C=O and –OH functional groups and a decrease in C–O–C groups, the adsorption capacity of KOH-BC300 for Cd(II) and Pb(II) was significantly enhanced. It was concluded that biochar’s adsorption performance might be greatly improved by increasing its oxygen-containing functional groups and specific surface area, but the effect of oxygen-containing functional groups was greater than that of specific surface area. Thus, KOH-modified biochar can be used as an effective sorbent to remove heavy metals from water.

## 3. Materials and Methods

### 3.1. Biochar Preparation

Pristine biochar was prepared in a continuous pyrolysis apparatus utilizing agricultural waste rice husk as the raw material. Rice husk was oven-dried at a temperature of 60 ± 1 °C until it reached a constant weight. It was then loaded into the alumina corundum semicircle ark (100 × 20 × 15 mm) and pyrolyzed in a tubular furnace (JS-G4012DGW, Tianjin, China) with limited oxygen by introducing nitrogen gas. The nitrogen flow rate was maintained at 200 mL·min^−1^. The pyrolysis temperatures of 300 °C, 400 °C, and 500 °C were individually set with a heating rate of 10 °C·min^−1^. Each temperature was maintained for 2 h before cooling to room temperature. Subsequently, the materials were ground and sieved to obtain particles below 0.15 mm for the aging experiments. The samples were designated as BC300, BC400, and BC500.

### 3.2. Biochar Modification

HNO_3_ and KOH were selected as the modification reagents. An amount of 10.0 g pristine biochar was added to 300 mL of 25% HNO_3_ solution, and the solution was heated at 90 °C for 4 h using a thermostatic magnetic stirrer (DF-101S, Zhengzhou, China). Another 10.0 g of pristine biochar was immersed in 300 mL of KOH (2 mol·L^−1^) and stirred with a magnetic stirrer for 4 h at a speed of 300 rpm. After modification, the solid–liquid mixture was separated using a medium-speed qualitative filter paper (Φ11 cm), washed repeatedly with deionized water until the pH value of the filtrate reached neutral, and then dried in an oven at 25 °C until constant in weight. The samples treated with HNO_3_ were designated as HNO_3_-BC300, HNO_3_-BC400, and HNO_3_-BC500, while the samples treated with KOH were designated as KOH-BC300, KOH-BC400, and KOH-BC500.

### 3.3. Biochar Characterization

A thermogravimetric analyzer (TG) was used to analyze the rice husk biomass and that of its mixtures. The biochar’s elemental compositions were determined using elemental analysis (EA) (vario Micro cube, Frankfurt am Main, Germany). Following burning at 800 °C for 2 h, the weight loss of biochar was measured to determine the ash content. The equation O = 100 − (C + H + N+ ash) (wt%) was used to estimate the samples’ oxygen content. The nitrogen adsorption isotherms were measured at 77 K, and the specific surface area and pore size were determined by using adequate methods. The specific surface area was calculated by the BET equation based on the adsorption–desorption curve, with pore size distribution calculated via BJH. The total pore volume was determined by the single-point adsorption amount at P/P_0_ = 0.988, and the average pore size diameter was the average value of all pores. The surface morphologies were examined through scanning electron microscopy (SEM) (Quattro S, Thermo Fisher Scientific, Waltham, MA, America). Fourier-transform infrared spectroscopy (FT-IR) was utilized to identify the surface functional groups of the unmodified and modified biochar samples. It was achieved by means of a Nicolet iN10 infrared microscope (Thermo Fisher Scientific). Spectra were at a resolution of 4 cm^−1^ and obtained in the range from 400 to 4000 cm^−1^. After mixing and grinding to make the samples less than 200 mesh, XPS analyses (PHI 5000 Versa Probe, Uivac-Phi, Chiba, Japan) were performed to give the C1s, O1s, and N1s spectra using a monochromatic Al-KαX-ray source and a spot size of ~1 mm in diameter. The binding energies were determined with reference to the C1s component (284.8 eV), and the background was linearly subtracted. All data were treated using XPSPEAK Version 4.0 and Origin 7.0 software. The Gaussian and Lorentzian ratio is 0.8 and the FWHM parameters are 1.1–2.5 eV.

### 3.4. Adsorption Experiment

Batch adsorption experiments were carried out in aqueous solutions containing Cd (II) and Pb (II) at a concentration of 1000 mg·L^−1^. The simulated wastewater was prepared using Pb(NO_3_)_2_, Cd(NO_3_)_2_·4H_2_O. The adsorption system’s pH was adjusted to 5.0 ± 0.2 with 1 mol·L^−1^ HNO_3_ or 1 mol·L^−1^ KOH solutions.

Bath adsorption studies were conducted to investigate the adsorption behaviors. For the adsorption kinetics, 0.25 g of unmodified or modified biochar was mixed with 100 mL of a 50 mg·L^−1^ Cd (II) or Pb (II) solution. Subsequently, the mixture was then subjected to agitation at a constant speed (180 rpm) for 1440 min at a temperature of 25 °C. After time ranges from 0 to 1440 min, samples were taken with a pipetting gun and filtered through a 0.22 μm nylon membrane filter (GE cellulose nylon membrane).

In the adsorption isotherm studies, 0.25 g of unmodified or modified biochar was mixed with 100 mL of solutions with varying initial concentrations ranging from 10 to 300 mg·L^−1^. Subsequently, the mixture underwent agitation at a constant speed (180 r·min^−1^) for 1440 min at a temperature of 25 °C. Samples were then taken with a pipetting gun and filtered through a 0.22 μm nylon membrane filter (GE cellulose nylon membrane). The concentrations of Cd (II) and Pb (II) post-adsorption were analyzed using inductively coupled plasma emission spectrometry (ICP–OES) with detection wavelengths of 214.44 and 226.50 nm for Cd (II) and 220.35 nm for Pb (II) and detection limits of 0.003 mg·L^−1^ and 0.05 mg·L^−1^, respectively. Duplicate experiments were conducted, and the average values were obtained.

The amount of adsorbed HMs at equilibrium was calculated using the following equation:

$$q\;=\;\left(C_{0\;}-{\;C}_{e}\right)\cdot V/m$$

where q represents the adsorbed amount at equilibrium (mg·g^−1^), C_0_ (mg·L^−1^) is the initial concentration of HMs in the solution, C_e_ (mg·L^−1^) is the equilibrium concentration of HMs, V (L) is the volume of the solution, and m (g) is the mass of the adsorbent.

The adsorption kinetics were simulated using the pseudo-first-order and pseudo-second-order kinetic equations, described as follows:

$$\mathrm{Pseudo}\text{-}\mathrm{first}\text{-}\mathrm{order}\;\mathrm{kinetic}\;\mathrm{model}\text{:}\;{Q_{t}=Q}_{e}\left(1-e^{-k_{1}t}\right)$$

$$\mathrm{Pseudo}\text{-}\mathrm{second}\text{-}\mathrm{order}\;\mathrm{kinetic}\;\mathrm{model}\text{:}\;\frac{t}{Q_{t}}=\frac{1}{Q_{e}t}+\frac{1}{k_{2}Q_{e}^{2}}$$

where Q_t_ and Q_e_ denote the quantity of adsorption at time t and equilibrium (mg·g^−1^). k_1_ and k_2_ are the rate constants for the pseudo-first-order and pseudo-second-order kinetic models.

The adsorption isotherms were modeled with the Langmuir and Freundlich isotherm models, described as follows:

$$\mathrm{Langmuir}\;\mathrm{adsorption}\;\mathrm{isotherm}\;\mathrm{model}\text{:}\;Q_{e}=\frac{K_{L}Q_{m}C_{e}}{1+C_{e}}$$

$$\mathrm{Freundlich}\;\mathrm{adsorption}\;\mathrm{isotherm}\;\mathrm{model}\text{:}\;Q_{e}=K_{F}{C_{e}}^{-n}$$

where Q_e_ is the adsorbed amount at equilibrium (mg·g^−1^; Q_e_ is the same as q), Q_m_ represents the maximum adsorption capacity of biochar (mg·g^−1^), and K_L_ is the Langmuir adsorption constant related to the free energy of adsorption (L·g^−1^). K_F_ (mg·g^−1^) is the Freundlich constant related to the adsorption capacity and n is an empirical parameter related to the adsorption intensity.

## 4. Conclusions

With an increase in pyrolysis temperature, the aromaticity of rice husk biochar increased while its polarity decreased. Concurrently, both the specific surface area and total pore volume of biochar increased significantly. HNO_3_ and KOH modification notably increased the O contents compared to unmodified biochar. KOH-modified biochar exhibited a lower O/C ratio than KNO_3_-modified biochar, indicating enhanced hydrophobicity and aromaticity in the KOH-treated material. FTIR and XPS analyses revealed that both HNO_3_ and KOH modification significantly changed the oxygen-containing functional groups in biochar, especially biochars prepared at lower pyrolysis temperatures, such as HNO_3_-BC300 and KOH-BC300. HNO_3_ modification introduced nitro and carboxyl groups on the surface of HNO_3_-BC300, increasing the number of ether bond functional groups, thereby enhancing the hydrophilicity of biochar. However, KOH modification increased the content of hydroxyl groups on KOH-BC300 and reduced the content of ether bond groups. At the same time, the modification of rice husk-derived biochars greatly enhanced their ability to absorb Cd(II) and Pb(II) from wastewater. Following HNO_3_ modification, the adsorption capacities for Cd(II) and Pb(II) increased by factors of 1.45–3.87 and 2.78–12.52, respectively, under conditions of pH 5.0 ± 0.2 and 298 K. In contrast, KOH modification enhanced the adsorption capacities for Cd(II) and Pb(II) by factors of 4.24–12.10 and 7.87–23.92, respectively. Notably, KOH-BC300 exhibited the highest adsorption capacities, reaching 72.14 mg·g^−1^ for Cd(II) and 170.84 mg·g^−1^ for Pb(II). These results demonstrate that KOH modification was more effective than HNO_3_ modification at enhancing the adsorption of Cd(II) and Pb(II) onto rice husk-derived biochar. In addition, the specific surface area and total pore volume of biochar increased significantly after HNO_3_ and KOH modification. It was concluded that biochar’s adsorption performance might be greatly improved by increasing its oxygen-containing functional groups and specific surface area, but the effect of oxygen-containing functional groups was greater than that of specific surface area. Therefore, KOH-modified biochar (KOH-BC300) can be used as an effective sorbent to remove heavy metals from wastewater. In addition, biochar prepared with rice husk has wide prospects, but most of the current research on biochar has been conducted in laboratories. The actual environment is more complex than that in the laboratory, and more field experiments need to be carried out.

## Figures and Tables

**Table 1:** Elemental contents of pristine and modified biochar.

Sample	Ash (wt.%)	C (%)	H (%)	N (%)	O (%)	H/C	O/C
BC300	26.19	46.96	3.50	0.55	22.79	0.89	0.36
BC400	31.69	48.42	2.54	0.57	16.79	0.62	0.26
BC500	35.81	55.05	1.93	0.79	6.42	0.42	0.09
HNO_3_-BC300	24.67	28.74	2.32	2.59	41.68	0.96	1.09
HNO_3_-BC400	28.68	37.44	2.02	2.68	29.18	0.64	0.59
HNO_3_-BC500	30.64	44.85	1.76	2.47	20.28	0.47	0.34
KOH-BC300	10.83	53.56	4.07	0.61	30.93	0.90	0.43
KOH-BC400	16.55	61.81	3.10	0.67	17.87	0.60	0.22
KOH-BC500	24.83	58.02	2.24	0.61	14.30	0.46	0.18

**Table 2:** BET surface area, total pore volume, and average pore diameter of pristine and modified biochar.

Sample	BET Surface Area (m^2^·g^−1^)	Total Pore Volume (cm^3^·g^−1^)	Average Pore Diameter (nm)
BC300	0.6	0.0089	61.50
BC400	5.4	0.0098	7.29
BC500	23.0	0.021	3.58
HNO_3_-BC300	119.1	0.14	4.81
HNO_3_-BC400	26.2	0.033	5.03
HNO_3_-BC500	11.1	0.013	4.66
KOH-BC300	3.0	0.0091	11.48
KOH-BC400	26.9	0.035	5.22
KOH-BC500	74.9	0.065	3.45

**Table 3:** C1s, O1s, and N1s bonding states and relative atomic percentages on the biochar surfaces.

Line	Binding Energy (eV)	Structure	Relative Atomic Percentage (%)								
			BC300	BC400	BC500	HNO_3_- BC300	HNO_3_-BC400	HNO_3_-BC500	KOH- BC300	KOH- BC400	KOH- BC500
C1s	284.8	C–(C,H), C=C	71.54	76.09	80.47	55.62	61.53	75.95	68.00	74.09	75.57
	285.8–286.2	C–(O,N)	18.30	15.15	12.22	25.06	17.12	11.14	25.32	17.58	12.63
	286.9–287.9	C=O; O–C–O	4.17	3.82	3.78	19.32	8.15	8.39	3.48	3.66	6.88
	288.6–289.2	O–C=O	5.98	4.94	3.53	0.00	13.20	4.52	3.20	4.67	4.92
O1s	531.2	O=C	2.41	10.23	2.79	29.03	0.04	3.80	8.67	8.07	2.63
	531.8	O=C–O–(C,H)	13.81	16.07	7.31	17.46	30.47	5.66	14.96	7.30	5.82
	532.6	C–O–C;C–OH	37.32	36.12	29.61	35.69	41.57	34.29	40.53	31.31	26.80
	533.4	O=C–O–(C,H)	46.46	37.58	60.29	17.82	27.92	56.24	35.84	53.31	64.75
N1s	399.9–400.1	N–C,N–C–COOH	82.85	77.69	90.38	47.16	46.47	46.26	/	/	/
	401.5–401.8	Ammonium N	17.15	22.31	9.62	28.17	11.66	10.85	/	/	/
	405.8–406.2	Nitrate N	0.00	0.00	0.00	24.67	41.87	42.89	/	/	/

**Table 4:** Kinetic parameters for adsorption of Cd (II) by pristine and modified biochar.

Metal	Sample	Pseudo-First-Order Kinetic Model			Pseudo-Second-Order Kinetic Model		
		Q_e,1_ $(\mathrm{mg}\cdot g^{-1}$)	k_1_ (min^−1^)	R^2^	Q_e,2_ $(\mathrm{mg}\cdot g^{-1}$)	k_2_ $(g\cdot (\mathrm{mg}\cdot \min^{-1}$))	R^2^
Cd(II)	BC300	2.58	0.25	0.9733	2.66	0.16	0.9937
	BC400	6.86	0.13	0.9439	7.22	0.03	0.9912
	BC500	3.93	0.13	0.9271	4.13	0.05	0.9792
	HNO_3_-BC300	16.83	0.43	0.9993	16.98	0.09	0.9999
	HNO_3_-BC400	15.23	0.33	0.9946	15.49	0.05	0.9994
	HNO_3_-BC500	4.81	0.04	0.9394	5.17	0.01	0.9819
	KOH-BC300	19.80	0.39	0.9984	20.02	0.06	0.9999
	KOH-BC400	19.61	0.44	0.9921	19.84	0.07	0.9977
	KOH-BC500	19.28	0.40	0.9977	19.46	0.06	0.9967

**Table 5:** Kinetic parameters for adsorption of Pb (II) by pristine and modified biochar.

Metal	Sample	Pseudo-First-Order Kinetic Model			Pseudo-Second-Order Kinetic Model		
		Q_e,1_ $(\mathrm{mg}\cdot g^{-1}$)	k_1_ (min^−1^)	R^2^	Q_e,2_ $(\mathrm{mg}\cdot g^{-1}$)	k_2_ $(g\cdot (\mathrm{mg}\cdot \min^{-1}$))	R^2^
Pb(II)	BC300	4.20	0.12	0.9906	4.37	0.05	0.9946
	BC400	12.51	0.57	0.9990	12.57	0.24	0.9993
	BC500	10.89	0.27	0.9723	11.19	0.04	0.9920
	HNO_3_-BC300	20.37	0.37	0.9991	20.61	0.05	0.9995
	HNO_3_-BC400	20.21	0.49	0.9951	20.39	0.09	0.9981
	HNO_3_-BC500	15.20	0.42	0.9922	15.37	0.08	0.9959
	KOH-BC300	20.07	0.44	0.9896	20.31	0.06	0.9952
	KOH-BC400	20.50	0.41	0.9973	20.72	0.06	0.9996
	KOH-BC500	19.88	0.48	0.9980	20.03	0.09	0.9994

**Table 6:** Isotherm parameters for adsorption of Cd(II) by pristine and modified biochar.

Metal	Sample	Langmuir Adsorption Isotherms Model			Freundlich Adsorption Isotherms Model		
		Q_m_	K_L_	R^2^	1/n	K_F_	R^2^
Cd(II)	BC300	5.96	0.0062	0.9230	0.54	0.18	0.9573
	BC400	6.87	0.13	0.9927	0.15	2.98	0.9543
	BC500	4.69	0.14	0.9886	0.17	1.84	0.9674
	HNO_3_-BC300	23.12	0.62	0.9264	0.15	11.20	0.9639
	HNO_3_-BC400	17.50	0.60	0.9725	0.13	9.29	0.9540
	HNO_3_-BC500	6.79	0.08	0.9624	0.18	2.46	0.9506
	KOH-BC300	72.14	1.31	0.9791	0.16	35.38	0.8141
	KOH-BC400	29.11	2.36	0.9809	0.12	16.58	0.9353
	KOH-BC500	35.87	1.71	0.9705	0.12	19.51	0.8597

**Table 7:** Isotherm parameters for adsorption of Pb(II) by pristine and modified biochar.

Metal	Sample	Langmuir Adsorption Isotherms Model			Freundlich Adsorption Isotherms Model		
		Q_m_	K_L_	R^2^	1/n	K_F_	R^2^
Pb(II)	BC300	7.14	0.06	0.9778	0.25	1.74	0.9404
	BC400	11.86	0.10	0.9921	0.22	3.47	0.9509
	BC500	9.98	0.012	0.9781	0.42	0.73	0.9651
	HNO3-BC300	89.46	0.20	0.9653	0.31	23.20	0.9623
	HNO3-BC400	71.52	0.21	0.9648	0.26	21.81	0.9557
	HNO3-BC500	27.77	0.20	0.9566	0.18	10.81	0.9406
	KOH-BC300	170.84	2.02	0.9788	0.11	114.80	0.9519
	KOH-BC400	93.58	0.26	0.9819	0.24	32.56	0.9069
	KOH-BC500	102.82	0.44	0.9822	0.24	38.87	0.8245

**Table 8:** Comparison of Cd(II) and Pb(II) adsorption parameters with those reported in the literature.

Raw Material	Pyrolysis Temperature	Modification Reagents	Target Pollutant	Adsorption Capacity (298 K)	Reference
Rice straw	500 °C	/	Cd(II)	5.55 mg·g^−1^	(Wang et al., 2019) [45]
Rice straw	120 °C	Citric acid	Cd(II)	8.14 mg·g^−1^	(Wang et al., 2019) [45]
Wheat straw	600 °C	KOH; Fe(NO)_3_	Cd(II)	31.89 mg·g^−1^	(Zhu et al., 2020) [46]
Rice husk	300 °C	KOH	Cd(II)	72.14 mg·g^−1^	This study
Douglas fir green wood chips	700 °C	KOH	Pb(II)	80 mg·g^−1^	(Herath et al., 2021) [47]
Rice husk	750 °C	NaOH; NaCl + KCl (1:1)	Pb(II)	110.22 mg·g^−1^	(Wu et al., 2022) [48]
Poplar saw dust	700 °C	/	Pb(II)	62.68 mg·g^−1^	(Cheng et al., 2021) [49]
Rice husk	300 °C	KOH	Pb(II)	170.84 mg·g^−1^	This study

## Data Availability

The authors declare that the data supporting the results of this study are available in this article and can be obtained from the corresponding author upon reasonable request.
